# Supplementary material for: Recovering Individual Emotional States from Sparse Ratings Using Collaborative Filtering
Source: Affect Sci. 2022 Nov 19;3(4):799–817. doi: 10.1007/s42761-022-00161-2 (PMC9743951; doi:10.1007/s42761-022-00161-2)
Supplement: Supplementary file 1 — (PDF 595 kb) [file 42761_2022_161_MOESM1_ESM.pdf]

Supplementary Materials

Figure S1
Affective Image Rating Recovery separately by basic emotion category.

Figure S2
Emotional Video Rating Recovery separately by movie.

Figure S3
MICE estimation failure for each dataset

Figure S4
Affective Image Rating Recovery with the inclusion of Non-negative Matrix Factorization trained via multiplicative updating.

Figure S5
Emotional Video Rating Recovery with the inclusion of Non-negative Matrix Factorization trained via multiplicative updating.

Figure S6
Social Decision Recovery with the inclusion of Non-negative Matrix Factorization trained via multiplicative updating.

Supplementary Methods
1. Non-negative Matrix Factorization with Multiplicative Updating

Figure S1

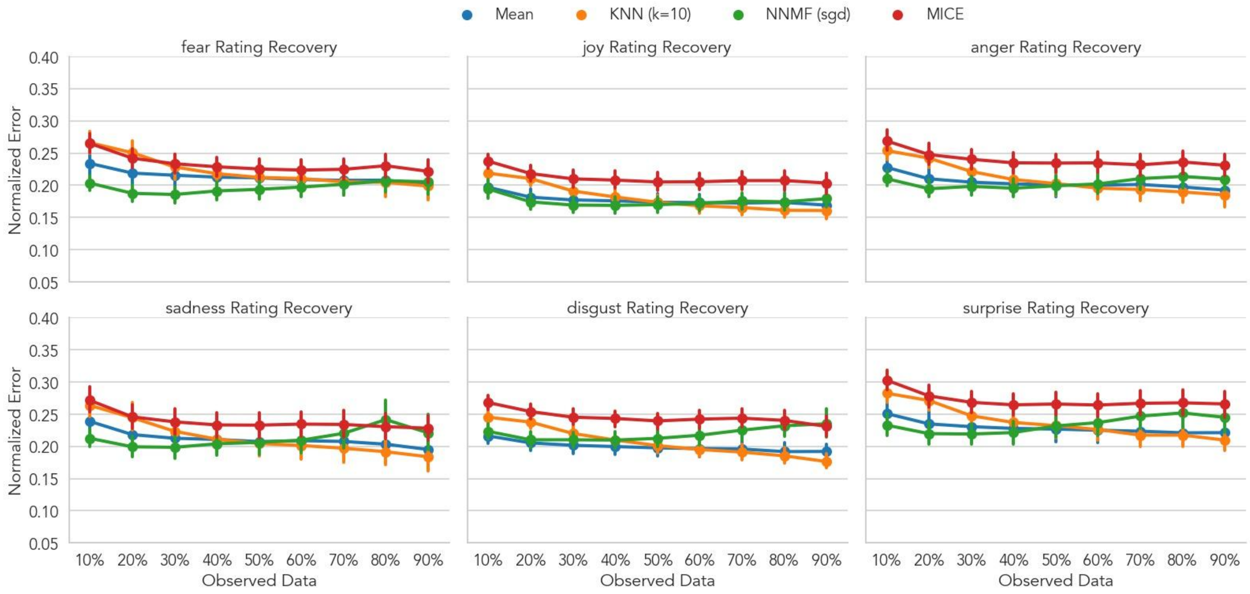

Affective image rating recovery separately by each basic emotion category
Each line depicts the cross-validated normalized RMSE averaged across users. Error bars depict 95% bootstrapped confidence intervals and points depict errors for individual users. Each facet depicts the performance of training and testing a model separately for each emotion category. Each emotion category showed largely consistent performance with the overall affective image recovery performance depicted in the main text (the average performance across all facets).

Figure S2

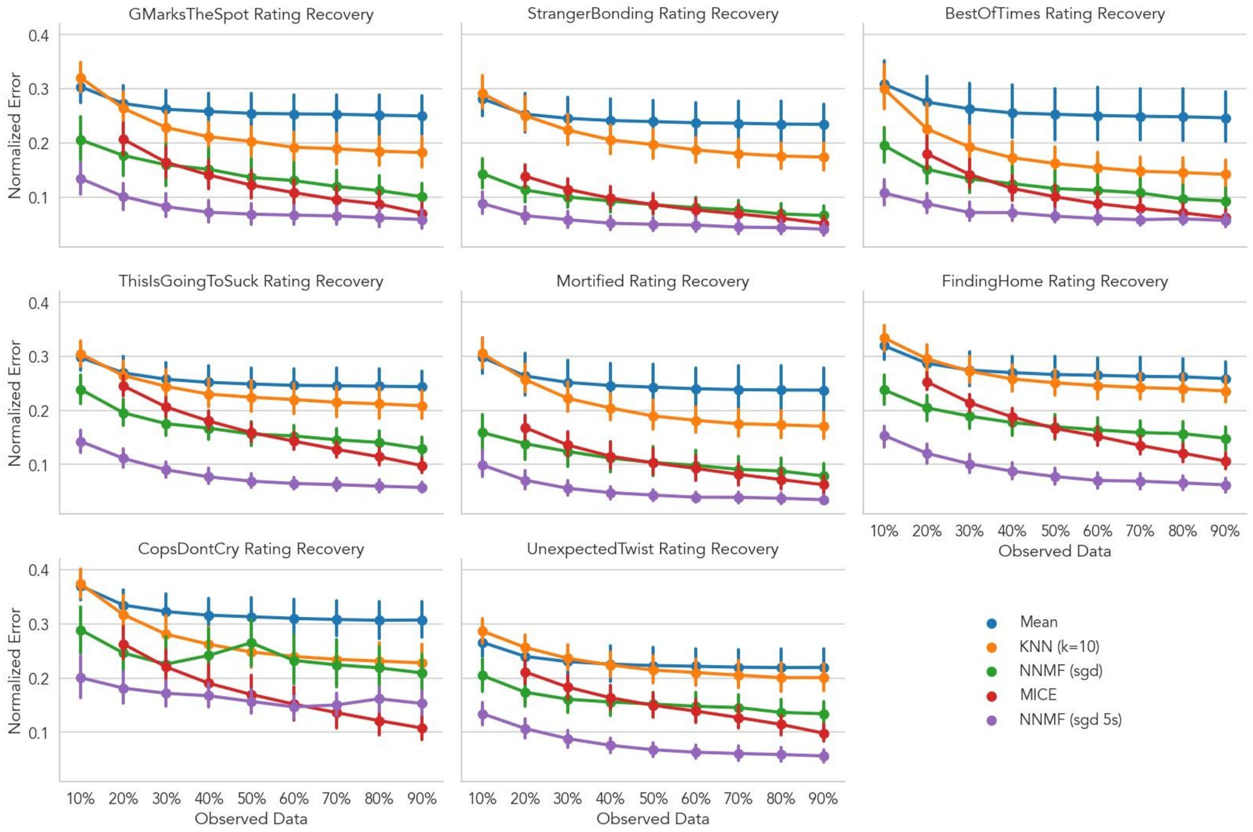

Emotional video rating recovery separately by each movie

Each line depicts the cross-validated normalized RMSE averaged across users. Error bars depict 95% bootstrapped confidence intervals and points depict errors for individual users. Each facet depicts the performance of training and testing a model separately for each video. Facets are arranged by video length with the top left plot depicting the shortest video and the bottom right depicting the longest video. Model performance was largely consistent across all videos and consistent with the overall emotional video recovery performance depicted in the main text (the average performance across all facets).

Figure S3

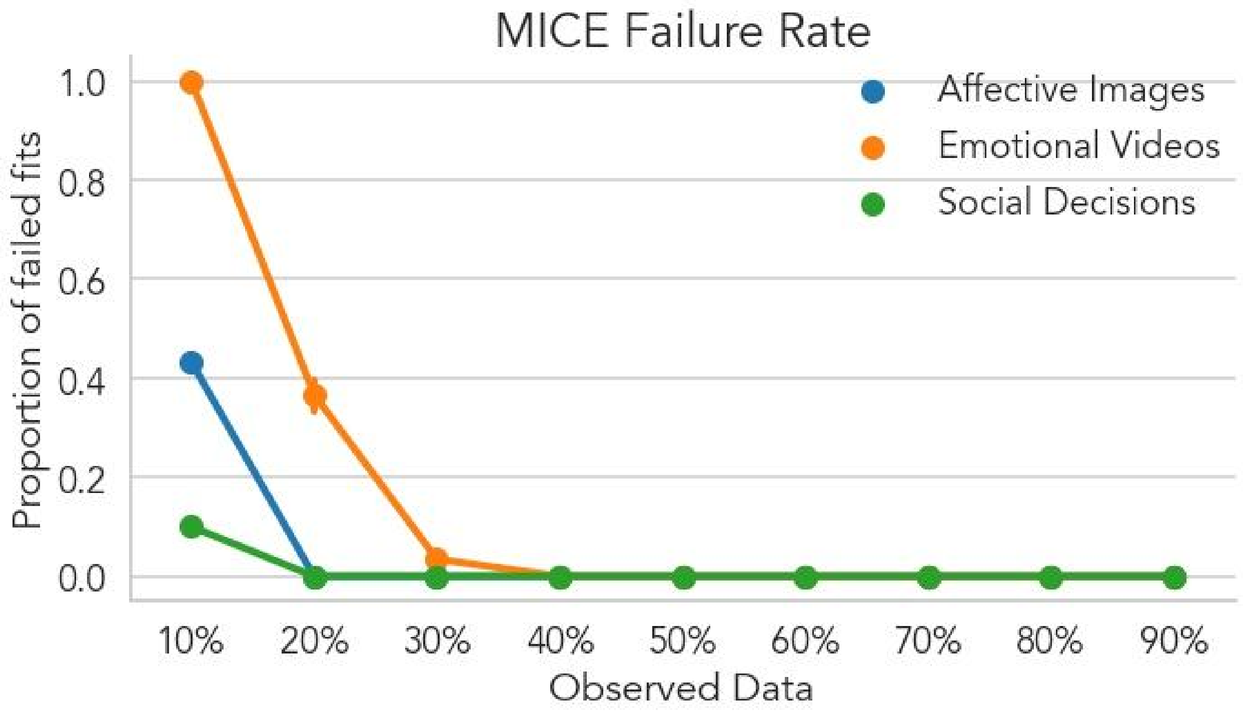

MICE Estimation failure rate

Average estimation for MICE across different levels of dataset sparsity. The y-axis depicts how many runs of MICE failed to estimate a model because no or not enough ratings were present for a given item to estimate its influence on a predicted item.

Figure S4

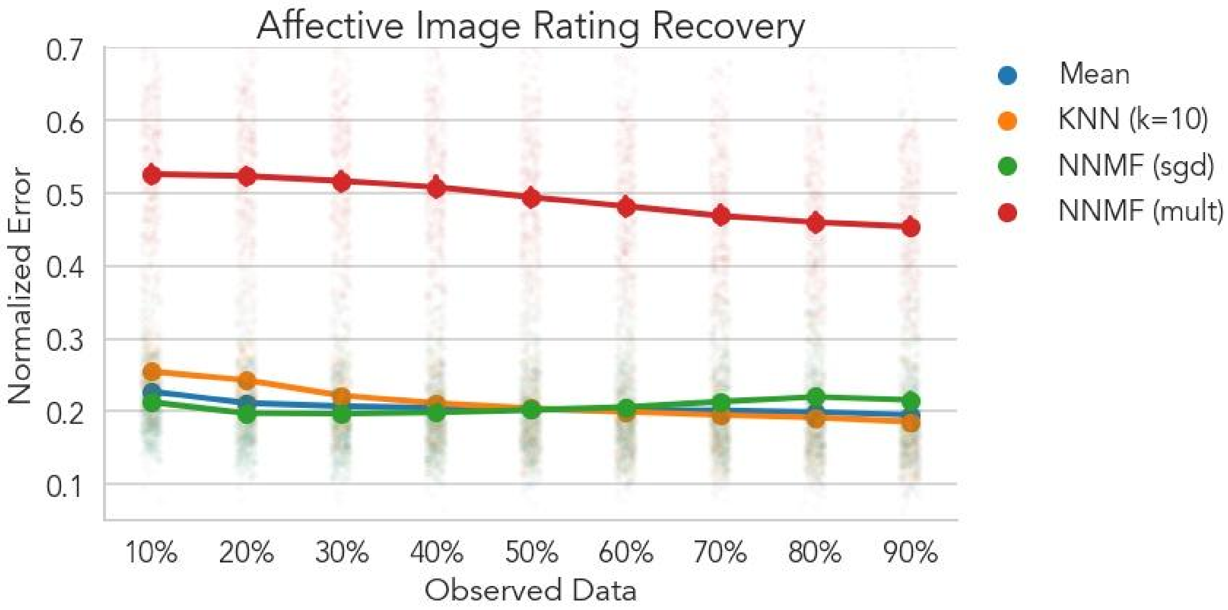

Affective image rating recovery including NNMF with multiplicative updating

Each line depicts the cross-validated normalized RMSE averaged across users. Error bars depict 95% bootstrapped confidence intervals and points depict errors for individual users. All algorithms except for NNMF multiplicative updating showed comparable performance in recovering user ratings to affective images regardless of dataset sparsity. Our implementation of multiplicative updating follows Lee & Seung's (2001) original implementation which doesn't explicitly handle sparsity. To address this missing ratings were replaced with 0s which likely lead to the poor out-of-sample performance.

Figure S5

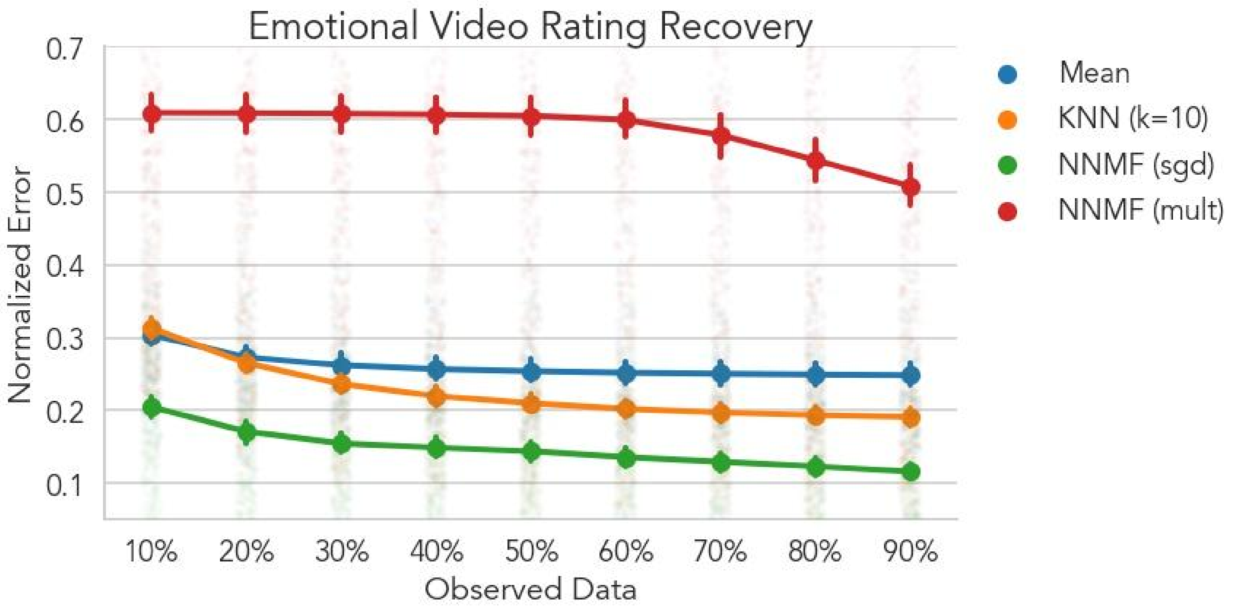

Emotional video rating recovery including NNMF with multiplicative updating
Each line depicts the cross-validated normalized RMSE averaged across users. Error bars depict 95% bootstrapped confidence intervals and points depict errors for individual users. NNMF trained with stochastic gradient descent outperformed all other algorithms at every level of dataset sparsity. KNN performed similarly to Mean imputation when data was especially sparse (<= 50% observed data%) due to issues computing reliable similarity estimates between users. However, as the amount of observed data increased, KNN performance exceeded Mean imputation.

Figure S6

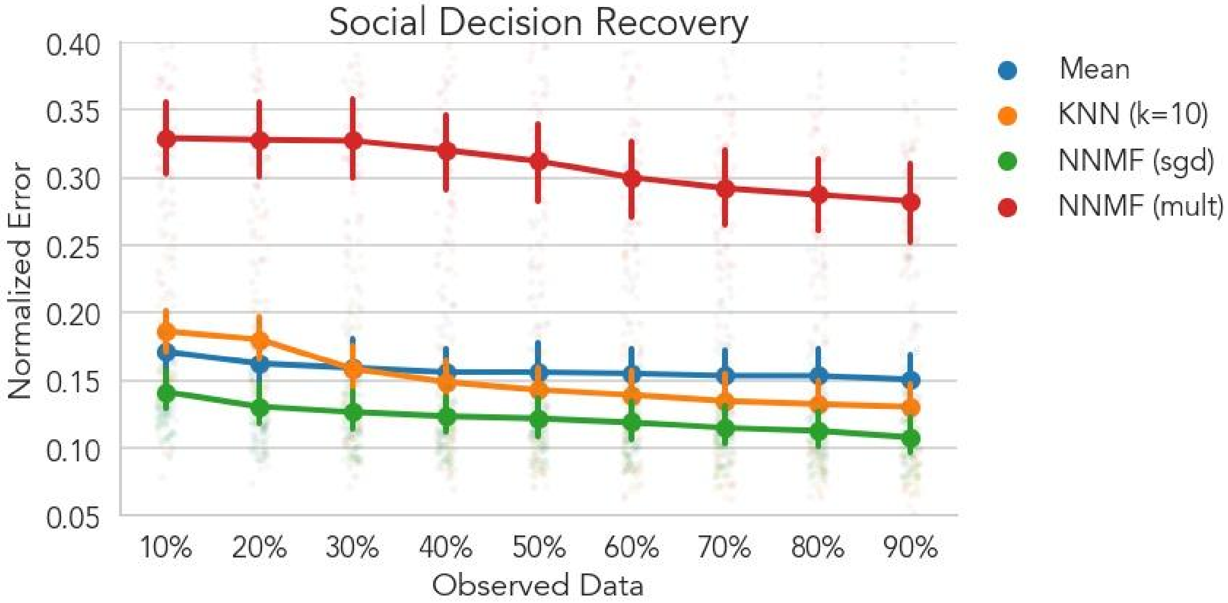

Social decision rating recovery including NNMF with multiplicative updating
Each line depicts the cross-validated normalized RMSE averaged across users. Error bars depict 95% bootstrapped confidence intervals and points depict errors for individual users. NNMF trained with stochastic gradient descent outperformed all other algorithms at every level of dataset sparsity. KNN performed similarly to Mean imputation when data was especially sparse (<= 50% observed data%) due to issues computing reliable similarity estimates between users. However, as the amount of observed data increased, KNN performance exceeded Mean imputation

Supplementary Methods

Non-negative Matrix Factorization with Multiplicative Updating

An alternative approach to learning matrix factors makes use of the multiplicative updating algorithm first proposed by Lee and Seung (2001). The model attempts to minimize the Euclidean distance (or Kullback-Liebler divergence) between the observed ratings in matrix V and the product of its factors WH:

$$L = ||V - WH||^2 \tag{Eq. 3}$$

The factor matrices are iteratively updated according to the following update rules:

$$H \leftarrow H \circ \frac{W^T V}{W^T W H} \tag{Eq. 4}$$

$$W \leftarrow W \circ \frac{V H^T}{W H H^T} \tag{Eq. 5}$$

However, this algorithm was not originally designed to handle cases with missing data, although several extensions have been made that attempt to facilitate this using a weighting matrix (Blondel et al. 2007). Our implementation closely follows the original algorithm, but prior to model fitting, unobserved values are set to 0 and updating continues until changes in loss fall below a specific threshold or a maximum number of iterations has been reached. As with the non-negative matrix factorization model trained via stochastic gradient descent, we arbitrarily selected 1e-6 as the error change threshold and 1000 iterations to ensure each fit ran for a similar amount while reducing overall computation time. Under multiplicative updating, the cost function L has been theoretically proven to be non-increasing with guaranteed convergence to local, but not globally optimal solutions (Lee and Seung 2001).

As depicted in Figures S4-S6 this approach performed worse than even the baseline model using mean imputation across all three datasets and is therefore a sub-optimal approach given the other algorithms discussed in the main text. Performance issues are likely because the algorithm treats 0 ratings as *true* ratings when learning matrix factors, thereby learning to reconstruct these values as if users deliberately made these ratings. This is supported by each supplementary figure which demonstrates how performance increases with decreasing sparsity, i.e. fewer 0 ratings that the model overfits to during training.
